# Sequencing and Partial Molecular Characterization of BAB-TMP, the Babeș Strain of the Fixed Rabies Virus Adapted for Multiplication in Cell Lines

**DOI:** 10.3390/v15091851

**Published:** 2023-08-31

**Authors:** Paulina Podgoreanu, Alexandru Petre, Radu Iulian Tănasă, Sorin Dinu, Mihaela Oprea, Ilinca-Mihaela Marandiuc, Ene Vlase

**Affiliations:** Cantacuzino National Military Medical Institute for Research and Development, 050096 Bucharest, Romania; podgoreanu.paula@yahoo.com (P.P.); petre.alexandru@cantacuzino.ro (A.P.);

**Keywords:** *Lyssavirus*, *Rhabdoviridae*, Babeș strain, rabies, ssRNA viruses, negative-sense ssRNA viruses, sequencing, genomics

## Abstract

The rabies virus induces a major zoonosis that causes severe nervous disease in humans, leading to paralysis and death. The world’s second anti-rabies center was established in 1888 by Victor Babeș, in Bucharest, where an eponymous strain of rabies was isolated and used to develop a method for immunization. The Babeș strain of the rabies virus was used for over 100 years in Romania to produce a rabies vaccine for human use, based on animal nerve tissue, thus having a proven history of prophylactic use. The present study aimed to sequence the whole genome of the Babeș strain and to explore its genetic relationships with other vaccine strains as well as to characterize its relevant molecular traits. After being adapted for multiplication in cell lines and designated BAB-TMP, 99% of the viral genome was sequenced. The overall organization of the genome is similar to that of other rabies vaccine strains. Phylogenetic analysis indicated that the BAB-TMP strain is closely related to the Russian RV-97 vaccine strain, and both seem to have a common ancestor. The nucleoprotein gene of the investigated genome was the most conserved, and the glycoprotein showed several unique amino acid substitutions within the major antigenic sites and linear epitopes.

## 1. Introduction

Rabies is an infectious disease caused by enveloped RNA viruses with neurotropic properties belonging to the order *Mononegavirales*, family *Rhabdoviridae*, genus *Lyssavirus* [1]. The infection is transmitted from animals to humans, especially through bites, and it is clinically manifested as acute encephalomyelitis, invariably fatal in unvaccinated persons [2,3]. Rabies is currently underdiagnosed and considered a neglected disease, despite the fact that approximately 59,000 people die from it annually, mostly in Africa and Asia. On average, one person dies of rabies every 10 min, mostly children from isolated and poor geographical areas, which places this disease as the main zoonosis worldwide [4,5,6,7].

In Romania, the virus is maintained in natural reservoirs represented by wild carnivores (mainly red foxes), which further infect domestic animals (dogs, cats), that can easily transmit the virus to humans. Although the incidence of rabies in Romania has decreased significantly in recent years, due to the implementation of an effective surveillance and control program aiming to eradicate the virus through oral vaccination of wild carnivore populations, there are still up to hundreds of cases of infected animals reported every year [8].

Virions are helically symmetrical, bullet-shaped (130–180 nm/60–100 nm) and contain an unsegmented genome (~12 kb long) consisting of a single-stranded, linear, negative-polarity RNA molecule, which encodes five proteins called N (nucleoprotein), P (phosphoprotein), M (matrix protein), G (glycoprotein), and L (large RNA-dependent RNA polymerase) [9]. During viral replication, at the intracellular level, glycoprotein G (~ 65 kDa), the only surface protein, is assembled into trimers that form spicules on the surface of virions, being the main structure inducing neutralizing antibodies and cell-mediated immunity [10]. As a result, all current rabies vaccines mainly contain the G protein derived from the rabies virus in concentrated form.

Currently, worldwide, there are three types of technologies for the production of rabies vaccines for human use [11,12]:

(a) Vaccines prepared from nerve tissue obtained from laboratory animals (sheep, mice, rabbits) inoculated with fixed virus. The initial technology was established by Louis Pasteur at the end of the 19th century (starting from 1885), but this technology no longer complies with the current requirements of the WHO and is very rarely used in some countries in Africa and South America;
(b) Vaccines based on embryonated eggs (hen and duck) inoculated with fixed rabies virus were developed in the 1950s, having the disadvantage that they are less immunogenic;
(c) Vaccines prepared in cell cultures, which are based on the multiplication of the fixed rabies virus in vitro, and comply with the current requirements of the WHO; these are the only vaccines currently used in the countries of the European Union.

Anti-rabies therapy for human use was introduced in Romania by Victor Babeș and his collaborators as early as 1889, being among the first sero-vaccines of this type prepared in the world, contemporary with those prepared by Louis Pasteur [13,14,15]. The fixed Babeș strain was then used in Romania for the production of an inactivated rabies vaccine up to the mid-1990s, through the technology of preparation from nervous tissues harvested from laboratory rabbits, lambs, and neonatal mice [16].

However, according to the current WHO and EU Pharmacopoeia requirements, the rabies vaccines for human use are presently based on viral fixed strains multiplied in cell cultures, being the only ones currently used in EU/EEA countries. In this regard, starting with the year 2020, the Babeș strain of the fixed rabies virus was adapted for multiplication in cell cultures, paving the way for preparation of the rabies vaccine through modern, contemporary methods. 

In this paper, we present, for the first time, the genome sequence of the Babeș strain of the fixed rabies virus, adapted for multiplication in cell cultures and designated BAB-TMP, as well as its phylogenetic relationships to other classic vaccine strains, with particular focus on the G viral gene and protein.

## 2. Materials and Methods

### 2.1. Cell Lines and Virus

The mouse neuroblastoma C1300 clone NA cell line (ECACC, Catalogue no. 93120817) was maintained in RPMI 1640 medium (INSTAMED, Biochrom AG, Berlin, Germany) supplemented with 10% fetal bovine serum (Gibco, Life Technologies Corporation, Paisley, UK) and antibiotics (PenStrep and Amphotericin B, Biochrom GmbH/Merck, Berlin, Germany).

The baby hamster kidney BHK-21 clone 13 cell line (ATCC, CCL-10) was maintained in Dulbecco’s modified Eagle’s medium/Ham’s nutrient mixture F-12 (DMEM/F-12, Sigma-Aldrich CHEMIE GmbH, Steinheim, Germany) supplemented with 10% fetal bovine serum (Gibco, Life Technologies Corporation, Paisley, UK) and antibiotics (PenStrep and Amphotericin B, Biochrom GmbH/Merck, Berlin, Germany).

The Babeș strain of the rabies virus was originally isolated by Professor Victor Babeș from a rabid wolf in the Bucharest region, Romania, back in 1887, and has since been maintained through intracerebral (i.c.) passages in rabbits. In this study, the viral strain was isolated and adapted for multiplication in cell lines after 3891 passages in laboratory rabbits plus 1 passage in Swiss/NMRI mice, followed by 14 passages in cell cultures. Primary virus isolation was carried out in C1300-NA cells using cerebral tissues from inoculated mice, mainly using the standard methods [17,18]. After 5 passages in C1300-NA and 5 passages in BHK-21 cells, the virus was pseudo-cloned once, through limited dilution, for enhanced cytopathogenicity and renamed BAB-TMP (Babeș–Tanasa, Marandiuc, Podgoreanu). Then, the strain was further multiplied in the BHK-21 cell line, and during cell passage 14 (5 passages in C1300 cells + 9 passages in BHK-21 cells), it was used for genome sequencing. Identification of the rabies virus multiplication in cell lines was performed using a direct immunofluorescence assay (DFA), using commercial specific FITC-conjugated polyclonal (BIO-RAD, ref. 3572112) and monoclonal (FUJIREBIO Diagnostics, ref. 800–092) anti-rabies virus antibodies, according to instructions.

### 2.2. Viral Genome Amplification

Viral RNA was extracted from 100 µL of cell culture supernatant using the Quick-RNA Viral Kit (Zymo Research, Irvine, CA, USA), eluted in 15 µL of nuclease-free water, and stored at −70 °C. Complementary DNA was synthetized using 5 µL of viral RNA with a ProtoScript First Strand cDNA Synthesis Kit and random hexamer primer (New England Biolabs, Ipswich, MA, USA). 

Pan-lyssavirus degenerate primers [19] and custom-designed primers were used to amplify overlapping fragments spanning the entire genome of the Babeș strain (Table 1).

PCR reactions carried out with the pan-lyssavirus primers were performed using 1.25 U of GoTaq G2 Hot Start Taq (Promega) DNA polymerase, 10 µL of 5× Green Flexi Buffer, 1.5 mM MgCl_2_, 0.2 mM of each dNTP, 1 µM of each primer pair, 5 µL of viral DNAc template, and nuclease-free water to a final volume of 50 µL. PCR amplifications with primers N127 and N8m were carried out on a BioRad T100 Thermal Cycler and consisted of 1 cycle at 94 °C for 2 min, followed by 40 cycles of 94 °C for 30 s, 51 °C for 60 s, and 72 °C for 90 s, with a final cycle at 72 °C for 5 min. For the amplicons obtained with the primers N1304-S2–G3393-AS2, G4836-S3–L7386-AS3, Taq3long–L9633-AS3, and L9267-S2–L11872-AS2, thermocycling conditions consisted of initial denaturation at 94 °C for 2 min, followed by 40 cycles of 94 °C for 30 s, 45 °C for 60 s, and 72 °C for 2.5 min, with a final extension of 72 °C for 5 min.

With the custom-designed primers (BAB-2964S and BAB-5690AS), PCR reactions were performed using 1 U of Phusion Hot Start II (New England Biolabs, Ipswich, MA, USA) DNA Polymerase, 10 µL of 5× Phusion Green Buffer, 0.2 mM of each dNTP, 0.5 µM for each primer pair, 1 µL of viral DNAc template, and nuclease-free water to a final volume of 50 µL. Initial denaturation was set at 98 °C for 30 s, followed by 35 cycles of 5 s denaturation at 98 °C, 20 s annealing at 51 °C, and 45 s extension at 72 °C, followed by a final extension at 72 °C for 7 min.

The PCR products were electrophoresed in a 1.5% agarose gel and stained with ethidium bromide solution. Molecular weight was estimated using the 1 kb DNA ladder (Promega), and the gels were visualized using Bio-Rad Gel Doc XR+.

Prior to sequencing, the PCR products were purified with the Wizard SV Gel and PCR Clean-up System (Promega). 

### 2.3. Sequencing and Phylogenetic Analysis

The purified amplicons were quantified (Qubit™ dsDNA HS kit, Thermo Fisher Scientific, Waltham, MA, USA), pooled in equimolar amounts, and used for DNA library preparation (400-base read length chemistry) with an Ion Xpress Plus Fragment Library Kit and Ion Xpress Barcode Adapters Kit, Thermo Fisher Scientific. After library size selection (E-gel SizeSelect II Agarose Gel 2%, Thermo Fisher Scientific), templates were prepared by using an Ion PGM Hi-Q View OT2 Kit and the Ion OneTouch 2 system (Thermo Fisher Scientific). Sequencing was performed on an Ion Torrent PGM platform using Ion 318 chip v2 BC and an Ion PGM Hi-Q View Sequencing Kit (Thermo Fisher Scientific). 

Reads were mapped against the NCBI Reference Sequence NC_001542.1 using Bowtie 2 Galaxy version 2.3.4, and the consensus sequence was called using ivar consensus (-q 20, -t 0.5, -m 10) [20]. The number of mapped reads was 184408, with an average base coverage depth of 3580X. For the phylogenetic analysis, we used the Molecular Evolutionary Genetics Analysis (MEGA) software ver. 11. The MEGA software was also used to determine percent homology between nucleotide or amino acid sequences.

Thirteen rabies virus full-length sequences used in the production of animal and human vaccines published in GenBank were included in the phylogenetic analyses: Pasteur, SAD B19, SAG 2, SRV9, CVS-11, CTN181, Flury-HEP, Flury-LEP, Nishigahara, RV-97, Pitman-Moore, ERA, and RC-HL (Table 2).

To test the effect of sequence variation on immunogenicity, we used the B-cell epitope prediction tool from IEDB, which employs the Bepipred 2.0 algorithm to assess linear epitopes. Each region was assessed independently taking into account its stereochemical conformation and we included the immunogenic region and its bordering sequence up until the closest alpha helix or beta sheet structure. The algorithm assigns a score to the inputted sequence, where the residues with scores above the 0.5 threshold are predicted to be part of an epitope.

In order to highlight possible changes in the main determinants of protective immunity, thirty-nine street strains of the rabies virus published in GenBank were used for the analysis of glycoprotein G sequences in relation to the BAB-TMP strain. These street strains were isolated from naturally infected animals in Romania and the rest of Europe [21].

## 3. Results

**Figure 1:**
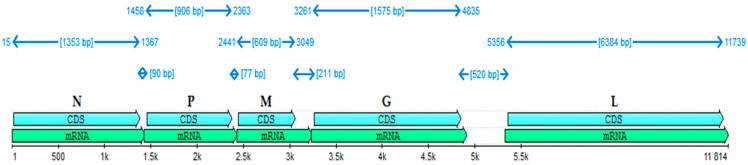
The organization of the rabies BAB-TMP genome, relative size of coding sequences (shown in blue), intergenic regions, mRNAs (shown in in green).

The almost full-length genome of the BAB-TMB strain was deduced by sequencing the appropriate PCR fragments and mapping the reads against the NCBI RABV reference. The overall result has 11,814 nucleotides, meaning 99% of the viral genome. The N gene (15–1367), P gene (1458–2363), M gene (2441–3094), G gene (3261–4835), and L gene (5356–11,739) identified in the BAB-TMB strain genome sequence are represented in Figure 1, along with the relative size of their corresponding mRNAs. At the nucleotide level, N was the most conserved among the five coding genes, indicating this gene is the most appropriate for quantitative genotype definition. We determined in silico that the N, P, M, G, and L proteins encoded by the analyzed genome contain 450, 301, 202, 524, and 2127 amino acid residues, respectively. 

The divergence between the whole genome of BAB-TMB and other rabies viruses ranges from 6.48% to 15.88%, which indicates that little nucleotide variation occurred. The strain that shows the highest degree of similarity is RV-97 (93.46%). The Russian vaccine strain RV-97 has been used for the oral live attenuated rabies vaccine for wildlife immunization [22]. 

**Figure 2:**
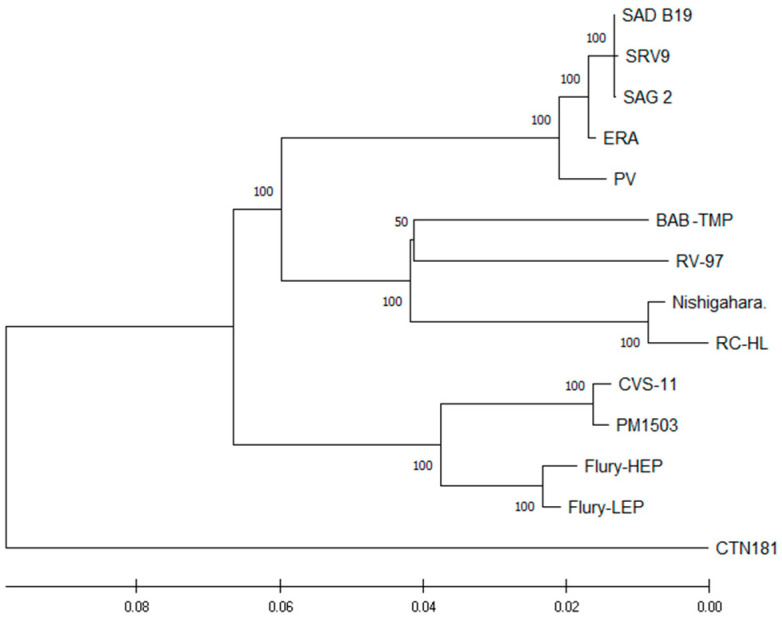
Phylogenetic tree of vaccine strains of *Lyssavirus*, constructed through the neighbor-joining method with 1000 bootstraps using whole genomes.

Comparison of the genomic sequences showed the biggest differences between the BAB-TMB strain and the CTN181 strain (84.12%), followed by the CVS-11 strain (89.93%) (Figure 2), at the nucleotide level.

**Figure 3:**
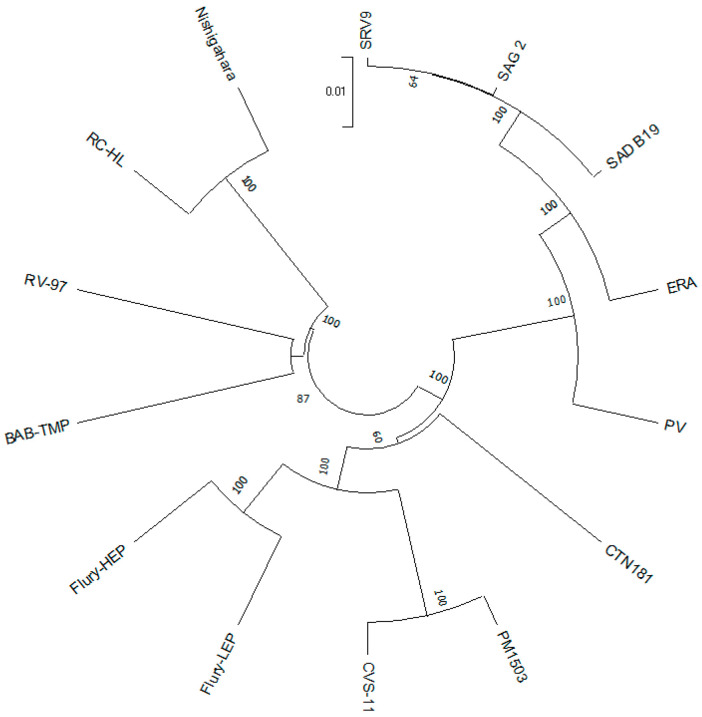
Phylogenetic tree of vaccine strains of *Lyssavirus*, constructed through the neighbor-joining method with 1000 bootstraps using whole proteomes.

The phylogenetic tree of whole proteome vaccine strains of *Lyssavirus* confirmed that the BAB-TMP strain is closely related to the RV-97 strain (Figure 3). The greatest differences were observed between the BAB-TMP strain and the SAD B19, SAG 2, SRV9, and RC-HL strains (Table 3). At the proteome level, there were no divergence values greater than 6.13% between BAB-TMB and other rabies viruses. The amino acid similarity is higher than the nucleotide similarity, which means that many of the nucleotide mutations are synonymous. We found the BAB-TMP N protein to be the most conserved, followed by L, P, G, and M (Table 3).

We compared the entire proteome of the BAB-TMP strain with the proteome of the RV-97 strain, which showed the closest phylogenetic relationship. After characterizing the nucleoprotein, we found a total of nine amino acid differences between the BAB-TMP and RV-97 strains. In the L protein, there were 80 differences, equally distributed along the protein sequence. In the P protein, 33 amino acids were different, and in the case of the M protein, there were 21 differences, distributed along the proteins. The glycoprotein had a total of 51 amino acid differences, which were located within the signal peptide region, ectodomain, trans-membrane domain, and cytoplasmic tail.

**Figure 4:**
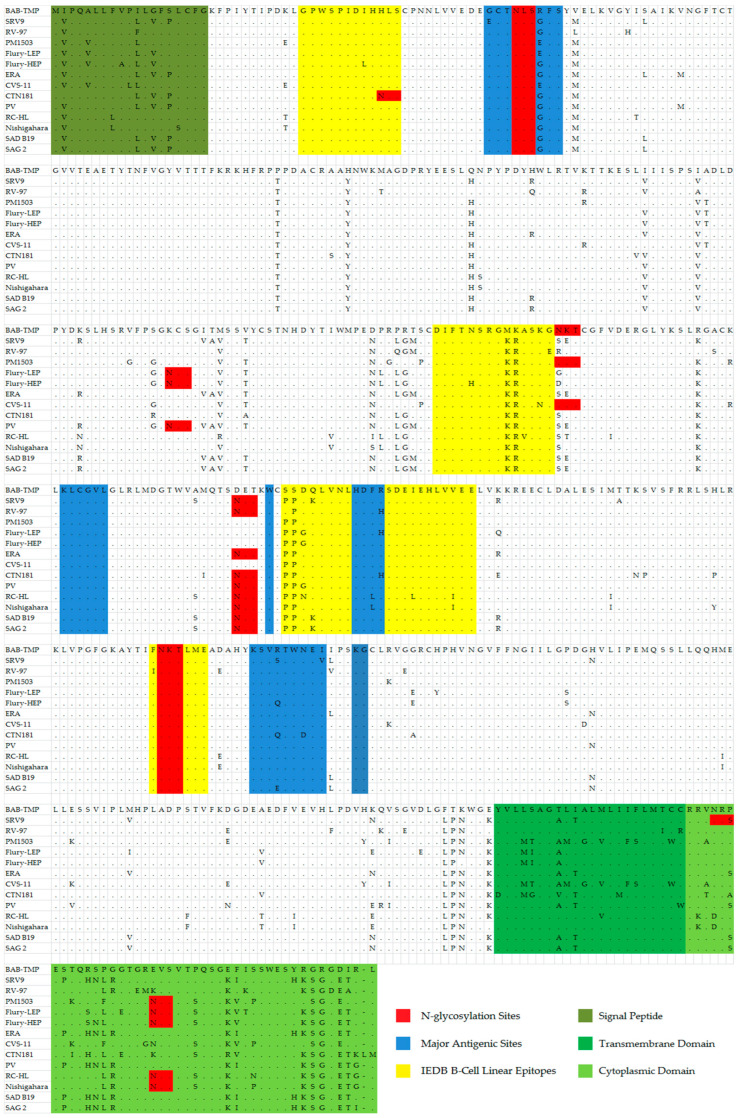
Amino acid comparison within the G gene among vaccine strains of rabies.

The viral glycoprotein G contains antigenic sites described in other studies [23,24] (Table 4). Antigenic site I (KLCGVL), IV (W), and site G1 (KG) display the same conserved sequence in the case of all analyzed vaccine strains. The antigenic site IIa presents a highly conserved sequence, with the exception of the BAB-TMP strain, that contains the amino acids Met-Lys-Ala instead of the conserved sequence Lys-Arg-Ala, a notable exception being RC-HL, where the last amino acid is Val. In the case of antigenic site IIb, the Babeș strain has Arg in position 40 instead of Gly, and this substitution is not found in any other vaccine strain among those analyzed in this study (Table 5). At this site, variation occurs at the Gly34 position for the SRV9 strain (Glu34) and Glu40 position for the PM1503, Flury-LEP, and CVS11 strains (Figure 4).

Regarding the antigenic site III (KSVRTWNEI), the BAB-TMP strain presents the same conserved sequence as the majority of the analyzed strains. The strains that show changes at this site are SRV9 (Ser333, Val338), Flury-HEP (Gln333), CTN181 (Gln333, Asp336), and SAG2 (Glu333). 

The G5 antigenic site presents the conserved HDFH sequence only in the RV-97, Flury-LEP, CTN181 strains, while the BAB-TMP strain has the HDFR sequence, respectively, where the last His amino acid is replaced with Arg.

The highly conserved epitope GPWSPIDIHHLS displays Leu19 instead of Ile for the Flury-HEP strain and His21 replaced by Asn for the CTN181 strain.

The highly conserved epitope DIFTNSRGKRASKG has His194 instead of Asn for the Flury-HEP strain, Val200 instead of Ala for the RC-HL strain, Asn202 instead of Lys for the CVS-11 stain, and Glu203 replacing Gly for the RV-97 strain. 

The epitope PPDQLVNLHDFRSDEIEHLVVEE displays a substitution unique for the BAB-TMP strain, where the two amino acids Pro253 and Pro254 usually found at the start of the sequence were each replaced by a molecule of Ser. Within the RV-97 strain, this substitution occurs only for Pro253. The major antigenic site G5 is contained within this B-cell neutralizing epitope. Other amino acid variations among the vaccine strains for this epitope are instead of Asp255 there is Gly (Flury-HEP and Flury-LEP, PV) and Asn (RC-HL); Gln256 substituted with Lys (SRV9, SAD B19, SAG 2); Ile268 replaced by Leu (RC-HL); and Val273 substituted by Ile (RC-HL, Nishigahara).

The FNKTLME epitope is found in the G protein and has the same sequence in all vaccine strains, the exception being the RV-97 strain, which contains Ile instead of Phe at the 318 position.

At the end of the G protein ectodomain, there is a substitution for three successive amino acids that is unique among the other vaccine strains: Phe434, Thr435, Lys436. The cytoplasmic domain of the BAB-TMP strain has certain amino acid substitutions found in no other vaccine strains analyzed in this paper, such as Pro474, Glu489, Gly499, Arg500, and Asp502.

When comparing the antigenic site/epitope sequence within the G gene among vaccine strains of rabies, we found significant variation only in certain regions, specifically antigenic sites IIb and III as well as the IEDB epitope 8644 and 48765.

The results showed that the Bepipred 2.0 algorithm assigned each of the assayed sequences a score above the detection threshold corresponding to the position of the known epitopes. None of the vaccine strains of rabies showed a significant decrease in immunogenicity based on the varying residue sequence. Accordingly, the unique substitutions of the BAB-TMP strain were not found to produce an immunogenic profile markedly different from other vaccine strains (Figure S1–S4).

To confirm that the BAB-TMP strain could be effective in providing protective immunity, we performed an amino acid comparison within glycoprotein G against street strains of rabies virus from Romania and the rest of Europe. Minor changes were identified in the major antigenic sites and in the epitope sequences (Table S1). Antigenic sites I and IV and epitopes ID 21838, 31772, and 17187 have highly conserved sequences. Street strains present the same highly conserved sequence (Lys-Arg-Ala) in the antigenic site IIa as in the case of most of the vaccine strains analyzed in this study, while the BAB-TMP strain contains the amino acids Met-Lys-Ala. In the antigenic site IIb, the BAB-TMP strain has Arg in position 40 instead of Gly, the latter being present both in isolates from Romania and in most vaccine strains. The rabies virus strains from Romania and Estonia have Val338 in antigenic site III, while the BAB-TMP strain and the rest of the vaccine strains have Ile338 in this position. While among the vaccine strains, the sequence of the antigenic site G1 remains conserved, isolates from Romania and Estonia substitute Lys for Arg at the 342 position. 

The street strains from Romania and the rest of Europe as well as the BAB-TMP strain present, with a few exceptions, the same sequence for the G5 antigenic site. The unique substitutions of the BAB-TMP strain are found in the epitope 48765, wherein the Ser253 and Ser254 differ from any street strain included in this study, as they have the conserved Pro254 and Pro254 variant similar to most vaccine strains.

It is thought that the modified level of N-glycosylation of the G glycoprotein is likely related to the adaptation of street rabies virus in cell culture and attenuation of its pathogenicity [25]. After examining the amino acid sequence of the G glycoprotein from the BAB-TMP strain, the number and positions of potential N-glycosylation sites were highlighted and are presented in Figure 4. The BAB-TMP strain has three potential sites at positions 37, 204, and 319 on the G protein, as in most fixed strains.

## 4. Discussion

In this study, we determined for the first time the genome sequence of the Babeș strain of the fixed rabies virus, which has been adapted for multiplication in cell lines. We also analyzed the phylogenetic relations of the BAB-TMP genome to other vaccine strains of rabies. We also mapped the position and modification of various B-cell linear epitopes and major antigenic sites for the BAB-TMP and other vaccine strains.

The isolated rabies virus (the street strain) was fixed by Professor Babeș through successive intracerebral passages in laboratory rabbits, being later used for the production of a rabies vaccine vaccine [13]. According to the practices of the time, viral antigens were produced through inoculation in laboratory animals (rabbits, lambs, mice), then the vaccine was prepared from nerve tissue by inactivating the virus with heat (37 °C) and phenol (until 1955) then through the Fermi method (phenol inactivation) until the mid-1970s [16]. Starting with 1974, the inactivated rabies vaccine was prepared by multiplying the virus in neonatal mouse brain followed by inactivation of the virus with ß-propiolactone, in order to obtain a purified preparation without myelin [14]. Between 1945 and the mid-1990s, the only human vaccine producer in Romania was the Cantacuzino Institute. Victor Babeș successively passed on the isolated strain through intracerebral inoculation in laboratory rabbits, weighing 1500–2000 g, so that in the first stage (1894–1928), the viral strain began to be fixed, having an incubation period of 5 days. Then, in the period between 1928 and 1938, the incubation period of the disease was invariably reduced to 4 days, followed by clinical signs of the nervous system and death of the inoculated animals after another 1–2 days [15].

The results of genome-wide phylogenetic analysis indicate that the BAB-TMP strain is closely related to RV-97, obtained from the Federal Center for Animal Health, Vladimir, Russia [22] (Figure 2). The complete genome sequences of the BAB-TMP and RV-97 strains have as high as 94.67% homology at the amino acid level, strongly suggesting that the two strains share a common ancestor. Furthermore, phylogenetic analysis of the rabies virus showed that isolates from Central–Eastern Europe have a strong geographical clustering, suggesting that there is some degree of genetic isolation [21], and that the strains isolated in Romania genetically clustered with either viruses from northeast Europe or with viruses circulating in the southwest of Russia [26]. On the other hand, the official history for the vaccine strain RV-97 is that it originated in the Pasteur strain (PV), but such an assumption was found to be unlikely on the basis of data published by Metlin et al. [22]. Another hypothesis could be that the Babeș strain represents the point of origin for the RV-97 strain. Perhaps within the emerging field of microbiology (represented by the prominent scientists L. Pasteur, V. Babeș, and E. Metchnikoff), towards the end of the 19th century, the first rabies vaccine strains were sometimes shared among researchers. Therefore, the Babeș strain might have been passed to Russian scientists to produce a rabies vaccine and implement the “Pasteur treatment” in Odessa, starting in 1886 [27].

Considering the amino acid differences among the five proteins, N was the most conserved (97.72%), followed by L (95.89%), P (90.22%), G (89.08%), and M (87.43%). The impact of these mutations that cause differences in the structures and functions of the individual proteins, and additionally inn viral phenotypes, have effects that remain unknown.

Generally, the G and N proteins are considered to be the most important determinant of the rabies virus for immunogenicity. At the nucleoprotein level, the amino acids Phe273 and Tyr394 are important for viral pathogenicity and in the mediated innate immunity of several fixed rabies virus strains [28]. Phe273 and Tyr394 were conserved in the BAB-TMP strain, but also in the other analyzed vaccine strains. The RC-HL strain is the only one that presents His394 instead of Tyr394.

The glycoprotein G plays an Important role in viral pathogenicity [29], being composed of a signal peptide region (amino acid position −19 to −1), ectodomain (1 to 439), trans-membrane domain (440 to 461), and cytoplasmic domain (462 to 504) [30]. Across all strains, the G protein retained the well-characterized antigenic sites II and III, a linear epitope located between residues 223–276, and an N-linked glycosylation site at residue 319 and residue Arg333 important for viral pathogenicity [31]. The importance of arginine or lysine at position 333 in the antigenic site III was proven in the studies of Tao et al. [32] and Takayama-Ito et al. [33]. Strains displaying other amino acid residues at this site cause nonlethal infections in adult mice following intracerebral inoculation [32,33]. The BAB-TMP strain presents the same conserved sequence and Arg333, as in the case of the majority of the analyzed strains. The strains that show changes in this position are SRV9 (Ser333) Flury-HEP (Gln333), CTN181 (Gln333), and SAG2 (Glu333). 

We compared the amino acid sequence identities within the rabies glycoprotein of the BAB-TMP strain to street strain isolates from Romania and the rest of Europe, with a focus on major antigenic sites and epitopes, resulting in a high degree of conservation for the primary targets for antibody recognition. We knew beforehand that the Babes vaccine strain is very efficient in providing protective immunity since it has been the only strain included in the preparation of vaccines for human use in Romania since more than 80 years ago. 

The National Institutes of Health (NIH) mouse test stands as a mandatory evaluation procedure for determining the potency of rabies vaccines intended for human use, particularly those prepared using cell cultures. This test ensures the vaccine’s effectiveness in inducing the desired immune response in vivo. Preliminary non-clinical trials have evaluated the potency of some vaccine candidate antigens prepared from the Babeș strain of fixed rabies virus adapted to the BHK-21 cell line. Vaccine antigens were prepared via inactivation with β-propiolactone and concentrated 11 times through tangential flow filtration. After inoculation, glycoprotein G-specific antibodies were quantified using ELISA with a commercial veterinary diagnostic kit (Platelia^TM^ Rabies II Kit, Bio-Rad, Hercules, CA, USA). The NIH assay revealed a relative potency (RP) of 2 IU/mL for the antigen. The sera of animals that resisted control infection had high concentrations of glycoprotein G-specific antibodies, greater than 4 EU/mL, with most having more than 200 EU/mL, 28 days after the boost. The outcomes revealed significant potency of the antigen obtained with tangential flow filtration, a scalable technology, which strongly justifies the progression of human rabies vaccine development toward validation and the initiation of pilot studies. Therefore, preliminary non-clinical data for immunogenicity testing using mice, according to WHO requirements, indicated that BAB-TMP is highly immunogenic as well [34].

An important role in the life cycle of viruses is played by potential N-glycosylation sites on the glycoprotein. In addition, the number and position of these sites determine the difference between the adaptability, pathogenicity, and antigenicity of street and fixed strains [35]. Street virus strains generally possess two sites for N-glycosylation in G protein, while laboratory-adapted strains present an additional site [36]. By increasing the number of glycosylation sites, the release of more infectious viruses is induced, which indicates that the release of the infectious virus may be an important factor in cell culture replication and adaptation of field viruses [25]. The BAB-TMP strain has three potential N-glycosylation sites at positions 37, 204, and 319 within the G protein, as in most fixed strains, which supports the hypothesis that virus production through increased G gene expression could play a major role in cell culture adaptation.

Our data suggest that the genome of the fixed BAB-TMP strain retained the basic characteristics of the rabies virus and is most closely clustered with the Russian rabies virus vaccine strain RV-97. Genetic knowledge can be of interest not only for the fundamental information that is necessary for the development of a therapeutic approach and a novel prophylaxis method for rabies in Romania, but it also provides new possibilities to investigate the impact of mutations of the whole proteome and linear B-cell epitopes and major antigenic sites of the BAB-TMP strain on virulence, pathogenicity, and induced immune response. 

## Figures and Tables

**Table 1:** List of primers used in this study.

Primer Name	Primer Sequence (5′ to 3′) ^1^	Position in the Reference Sequence ^2^
N127	F: ATGTAACACCTCTACAATGG	55–1586
N8m	R: CAGTCTCYTCNGCCATCTC	
N1304-S2	F: AAYGGRGGBMGAYTVAARAGATC	1304–3415
G3393-AS2	R: CADGGDCCNAGYTTGTCTGGTAT	
BAB-2964S	F: CGAGAGCATGTCAACTATGG	3019–5752
BAB-5690AS	R: CTGTTTGATTCAGAATGG	
G4836-S3	F: GGRARRGTYATATCTTCNTGGGA	4836–7411
L7386-AS3	R: CTRTCBGARTARTADAYCCANGACTT	
Taq3long	F: ATGAGAAGTGGAAYAAYCATCA	7273–9651
L9633-AS3	R: TGCYRTATATGTTGACAGG	
L9267-S2	F: ATGTTYCARCCNYTGATGCT	9267–11,901
L11872-AS2	R: AAAYAATCAADCARHYAGAGG	

^1^ F: forward, R: reverse; ^2^ reference sequence: GenBank acc. no. M13215.

**Table 2:** Sequences used in this study for the phylogenetic analyses.

Rabies Virus Vaccine Strain	Accession Number	Origin/ Derivation	Vaccine Type	Genome Length (bp)
Pasteur	M13215	France	Human	11,932
SAD B19	M31046	U.S.A./Germany	Animal	11,928
SAG 2	EF206719	U.S.A./France	Animal	11,928
SRV9	AF499686	China	Animal	11,928
CVS-11	GQ918139	France	Animal	11,927
CTN181	EF564174	China	Human	11,923
Flury-HEP	GU565704	U.S.A.	Human	11,925
Flury-LEP	DQ099524	U.S.A.	Human	11,711
Nishigahara	AB044824	Japan	Animal	11,926
RV-97	EF542830	France/Russia	Animal	11,932
Pitman-Moore	DQ099525	France/U.S.A.	Human	11,723
ERA	EF206707	U.S.A./Canada	Animal	11,931
RC-HL	AB009663	Japan	Animal	11,926

**Table 3:** Pairwise comparisons of predicted amino acid sequence identities of the BAB-TMP rabies virus with all five proteins and all coding sequences of other lyssaviruses.

Strains Compared with BAB-TMP	N (%)	P (%)	M (%)	G (%)	L (%)	All CDS (%)
Pasteur	97.33	90.94	88.57	88.74	95.42	94.17
SAD B19	97.78	90.27	86.67	89.12	95.53	93.98
SAG 2	97.78	90.27	86.67	88.74	95.58	93.95
SRV9	97.78	90.27	86.67	88.36	95.53	93.87
CVS-11	98.22	88.93	90.43	89.31	96.05	94.45
CTN181	97.11	87.25	91.90	88.17	95.96	94.06
Flury-HEP	98.44	87.58	91.43	90.08	96.29	94.67
Flury-LEP	98.44	87.58	91.90	89.31	96.10	94.48
Nishigahara	96.89	90.60	90.14	89.89	96.38	94.76
RV-97	98.00	89.23	90.00	90.27	96.24	94.67
Pitman-Moore	98.44	87.92	90.48	89.69	95.96	94.40
ERA	97.78	90.27	88.57	89.31	95.72	94.23
RC-HL	96.44	88.93	88.73	88.55	95.82	93.95

**Table 4:** Classification of neutralizing epitopes found within the rabies G protein.

IEDB Epitope			Major
Sequence	ID	Sequence	Antigenic Site
GPWSPIDIHHLS	21,838	-	-
-	-	GCTNLSGFS	IIb
DIFTNSRGKRASKG	8644	KRA	IIa
KLCGVL	31,772	KLCGVL	I
-	-	W	IV
PPDQLVNLHDFRSDEIEHLVVEE	48,765	HDFH	G5
FNKTLME	17,187	-	-
-	-	KSVRTWNEI	III
-	-	KG	G1

**Table 5:** Position and modifications of neutralizing sites for the BAB-TMP strain.

Position (BAB-TMP)	IEDB ID	Differences from IEDB Epitope Sequence	Major Antigenic Site Name	Differences from Major Antigenic Site Sequence
12–23	21,838	GPWSPIDIHHLS	-	-
34–42	-	-	IIb	GCTNLS[R]FS
190–203	8644	DIFTNSRG[MK]ASKG	IIa	[MK]A
226–231	31,772	KLCGVL	I	KLCGVL
250	-	-	IV	W
253–275	48,765	[SS]DQLVNLHDFRSDEIEHLVVEE	G5	HDF[R]
318–324	17,187	FNKTLME	-	-
330–338	-	-	III	KSVRTWNEI
342–343	-	-	G1	KG

Substituted amino acids for the BAB-TMP strain are shown within brackets.

## Data Availability

The BAB-TMP glycoprotein (G) sequence was submitted to the GenBank® database and can be retrieved under accession number OR500222.

## Supplementary Materials

The following supporting information can be downloaded at: https://www.mdpi.com/article/10.3390/v15091851/s1, Table S1: Amino acid variations of the antigenic determinants among street strains of the rabies virus from Romania and the rest of Europe compared to the BAB-TMP strain; Figure S1–S4: Linear B-cell epitope probability predicted with Bepipred 2.0 algorithm.
