# Supplementary material for: Sequencing and Partial Molecular Characterization of BAB-TMP, the Babeș Strain of the Fixed Rabies Virus Adapted for Multiplication in Cell Lines
Source: Viruses. 2023 Aug 31;15(9):1851. doi: 10.3390/v15091851 (PMC10536377; doi:10.3390/v15091851)
Supplement: Supplementary file 1 [file viruses-15-01851-s001.zip › viruses-2478154-supplementary.pdf]

**Supplementary appendix**

Table S1. Amino acid variations of the antigenic determinants among street strains of rabies virus from Romania and Europe compared to the BAB-TMP strain.

| Year | Lyssavirus rabies isolate | Accession number | Country | Amino acids substitutions compared to the BAB-TMP strain | | | | | | | | |
|---|---|---|---|---|---|---|---|---|---|---|---|---|
| | | | | IEDB Epitope ID / Antigenic site | | | | | | | | |
| | | | | 21838 | IIb | 8644 / IIa | 31772 / I | IV | 48765 / G5 | 17187 | III | G1 |
| 2022 | DR1017 | ULE27199 | Romania | - | Gly40 | Lys198, Arg199 | - | - | Pro253, Pro254 | - | Val338 | Arg342 |
| 2022 | DR1019 | UIG83415 | Romania | - | Gly40 | Lys198, Arg199 | - | - | Pro253, Pro254 | - | Val338 | Arg342 |
| 2022 | DR1021 | UIG83411 | Romania | - | Gly40 | Lys198, Arg199 | - | - | Pro253, Pro254 | - | Val338 | Arg342 |
| 2022 | DR1024 | UIG83431 | Romania | - | Gly40 | Lys198, Arg199 | - | - | Pro253, Pro254 | - | Val338 | Arg342 |
| 2022 | DR1025 | UIG83419 | Romania | - | Gly40 | Lys198, Arg199 | - | - | Pro253, Pro254 | - | Val338 | Arg342 |
| 2022 | DR1026 | UJD06171 | Romania | - | Gly40 | Lys198, Arg199 | - | - | Pro253, Pro254 | - | Val338 | Arg342 |
| 2022 | DR1027 | UJD06175 | Romania | - | Gly40 | Lys198, Arg199 | - | - | Pro253, Pro254 | - | Val338 | Arg342 |
| 2022 | DR1031 | UIG83407 | Romania | - | Gly40 | Lys198, Arg199 | - | - | Pro253, Pro254 | - | Val338 | Arg342 |
| 2022 | DR1034 | UIG83423 | Romania | - | Gly40 | Lys198, Arg199 | - | - | Pro253, Pro254 | - | Val338 | Arg342 |
| 2022 | DR1035 | UIG83435 | Romania | - | Gly40 | Lys198, Arg199 | - | - | Pro253, Pro254 | - | Val338 | Arg342 |
| 2022 | DR1036 | UIG83427 | Romania | - | Gly40 | Lys198, Arg199 | - | - | Pro253, Pro254 | - | Val338 | Arg342 |
| 2022 | DR1187 | UIG83443 | Romania | - | Gly40 | Lys198, Arg199 | - | - | Pro253, Pro254 | - | Val338 | Arg342 |
| 2022 | DR1198 | UIG83451 | Romania | - | Gly40 | Lys198, Arg199 | - | - | Pro253, Pro254, His264 | - | Val338 | Arg342 |
| 2022 | DR1200 | WAB53228 | Romania | - | Gly40 | Lys198, Arg199 | - | - | Pro253, Pro254 | - | Val338 | Arg342 |
| 2022 | DR1331 | WAB53233 | Romania | - | Gly40 | Lys198, Arg199 | - | - | Pro253, Pro254 | - | Val338 | Arg342 |
| 2022 | DR1333 | UIG83439 | Romania | - | Gly40 | Lys198, Arg199 | - | - | Pro253, Pro254 | - | Val338 | Arg342 |
| 2022 | DR1335 | UIG83447 | Romania | - | Gly40 | Lys198, Arg199 | - | - | Pro253, Pro254 | - | Val338 | Arg342 |
| 2022 | DR1345 | WAJ60187 | Romania | - | Gly40 | Lys198, Arg199 | - | - | Pro253, Pro254 | - | Val338 | Arg342 |
| 2022 | DR1348 | WAJ60183 | Romania | - | Gly40 | Lys198, Arg199 | - | - | Pro253, Pro254 | - | Val338 | Arg342 |
| 2022 | DR1351 | ULE27194 | Romania | - | Gly40 | Lys198, Arg199 | - | - | Pro253, Pro254 | - | Val338 | Arg342 |
| 2002 | 9202ALL | AAF36791 | Germany | * | * | Lys198, Arg199 | - | - | Pro253, Pro254 | - | - | - |
| 2002 | 9212ALL | AAF36790 | Germany | * | * | Lys198, Arg199 | - | - | Pro253, Pro254 | - | - | - |
| 2002 | 8903FRA | AAF36782 | France | * | * | Lys198, Arg199 | - | - | Pro253, Pro254 | - | - | - |
| 2002 | 9353FRA | AAF36780 | France | * | * | Lys198, Arg199 | - | - | Pro253, Pro254 | - | - | - |
| 2002 | 9427FRA | AAF36783 | France | * | * | Lys198, Arg199 | - | - | Pro253, Pro254 | - | - | - |
| 2002 | 94288FRA | AAF36784 | France | * | * | Lys198, Arg199 | - | - | Pro253, Pro254 | - | - | - |
| 2002 | 9429FRA | AAF36786 | France | * | * | Lys198, Arg199 | - | - | Pro253, Pro254 | - | - | - |
| 2002 | 9445FRA | AAF36785 | France | * | * | Lys198, Arg199 | - | - | Pro253, Pro254 | - | - | - |

| | | | | | | | | | | | | |
|---|---|---|---|---|---|---|---|---|---|---|---|---|
| 2002 | 9616FRA | AAF36781 | France | * | * | Lys198, Arg199 | - | - | Pro253, Pro254 | - | - | - |
| 2002 | 9244FRA | AAF36795 | France | * | * | Lys198, Arg199 | - | - | Pro253, Pro254 | - | - | - |
| 2002 | 9223FRA | AAF36779 | France | * | * | Lys198, Arg199 | - | - | Pro253, Pro254 | - | - | - |
| 2002 | 9215HON | AAF36796 | Hungary | * | * | Ile193, Lys198, Arg199 | - | - | Pro253, Pro254 | - | - | - |
| 2002 | 9384HON | AAF36793 | Hungary | * | * | Lys198, Arg199 | - | - | Pro253, Pro254 | - | - | - |
| 2002 | 86107YOU | AAF36798 | Yugoslavia | * | * | Lys198, Arg199 | - | - | Pro253, Pro254 | - | - | - |
| 2002 | 8653YOU | AAF36794 | Yugoslavia | * | * | Lys198, Arg199 | - | - | Pro253, Pro254, Gly255 | - | - | - |
| 2002 | 8658YOU | AAF36797 | Yugoslavia | * | * | Lys198, Arg199 | - | - | Pro253, Pro254 | - | - | - |
| 2002 | 9142EST | AAF36792 | Estonia | * | * | Lys198, Arg199 | - | - | Pro253, Pro254 | - | V-357 | Arg342 |
| 2002 | 9339EST | AAF36788 | Estonia | * | * | Lys198, Arg199 | - | - | Pro253, Pro254 | - | V-357 | Arg342 |
| 2002 | 9342EST | AAF36789 | Estonia | * | * | Lys198, Arg199 | - | - | Pro253, Pro254 | - | V-357 | Arg342 |

* = No Data

**Supplementary appendix:** Linear B-cell epitope probability predicted with Bepipred 2.0 algorithm

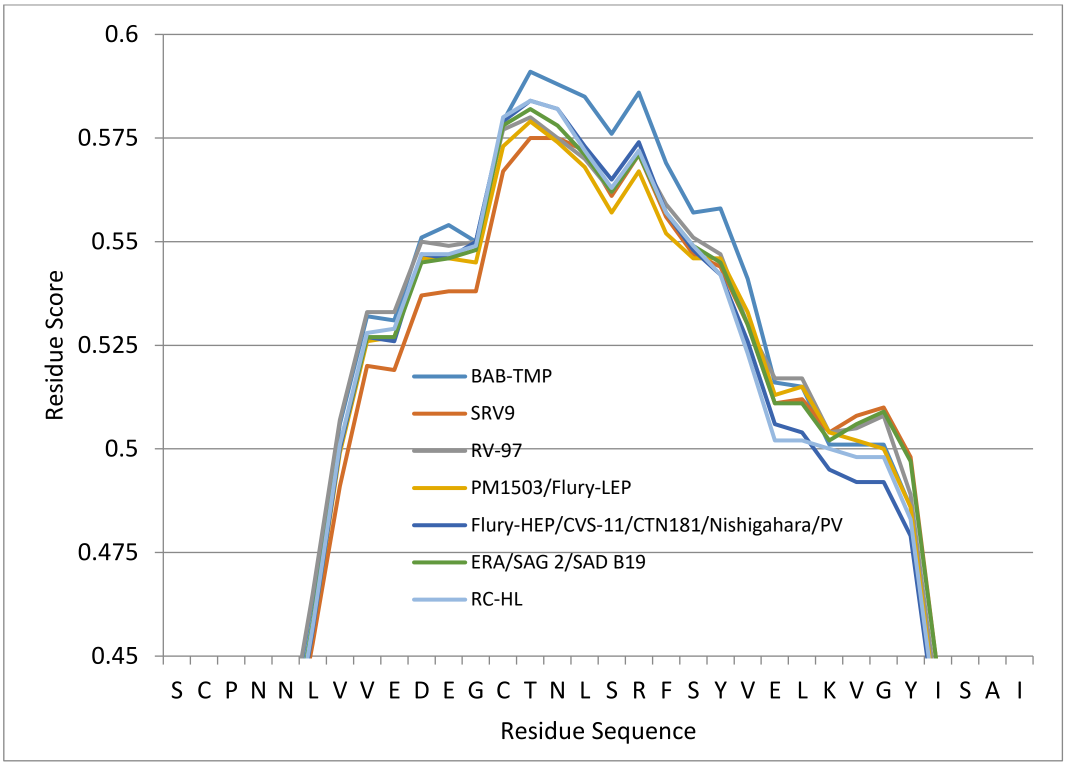


Figure S1. Linear B-cell epitope probability predicted with Bepipred 2.0 algorithm for Antigenic Site IIb

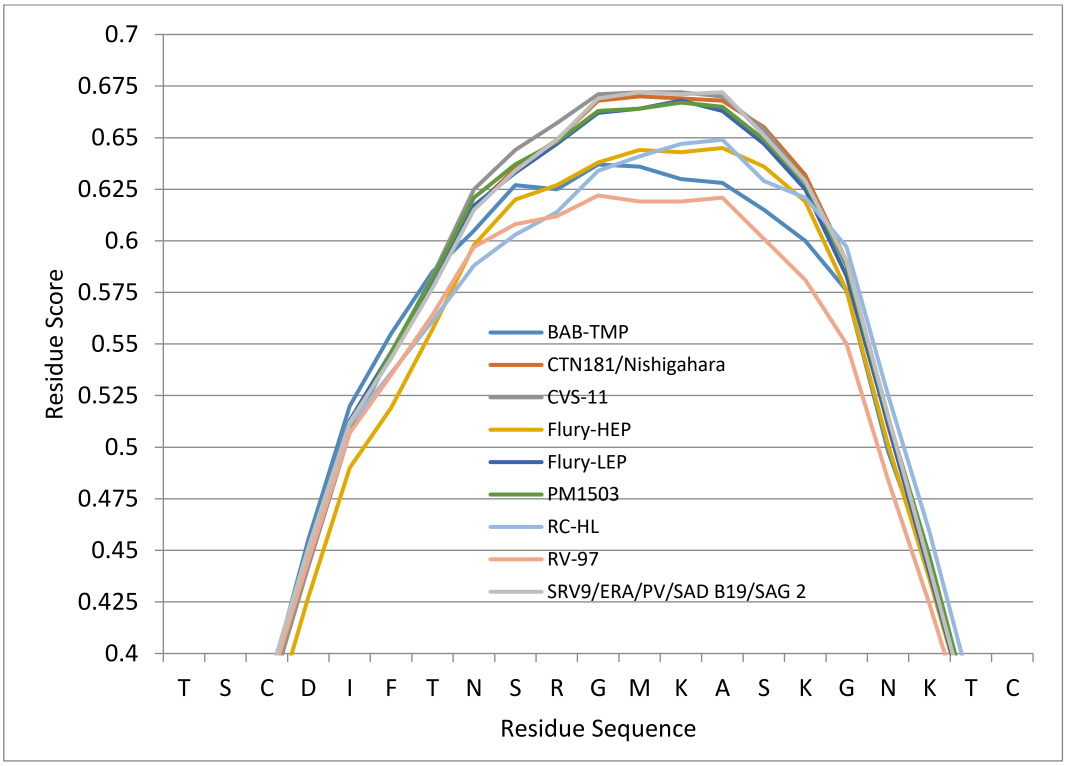


Figure S2. Linear B-cell epitope probability predicted with Bepipred 2.0 algorithm for IEDB ID 8644

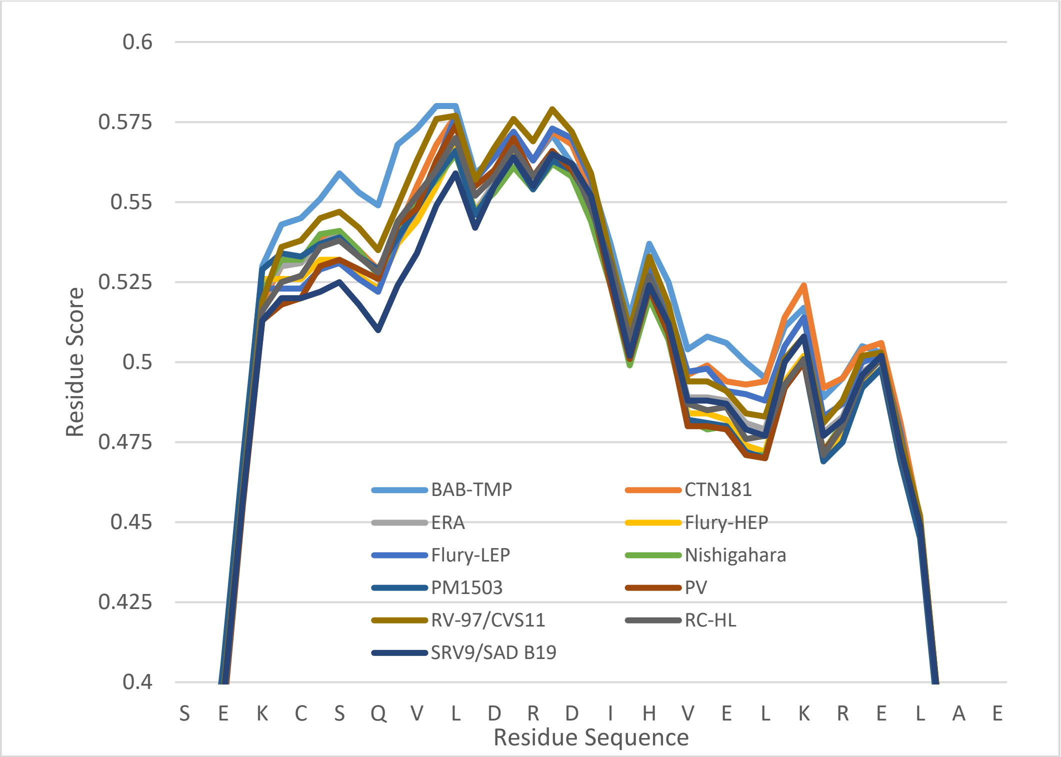


Figure S3. Linear B-cell epitope probability predicted with Bepipred 2.0 algorithm for IEDB ID 48765

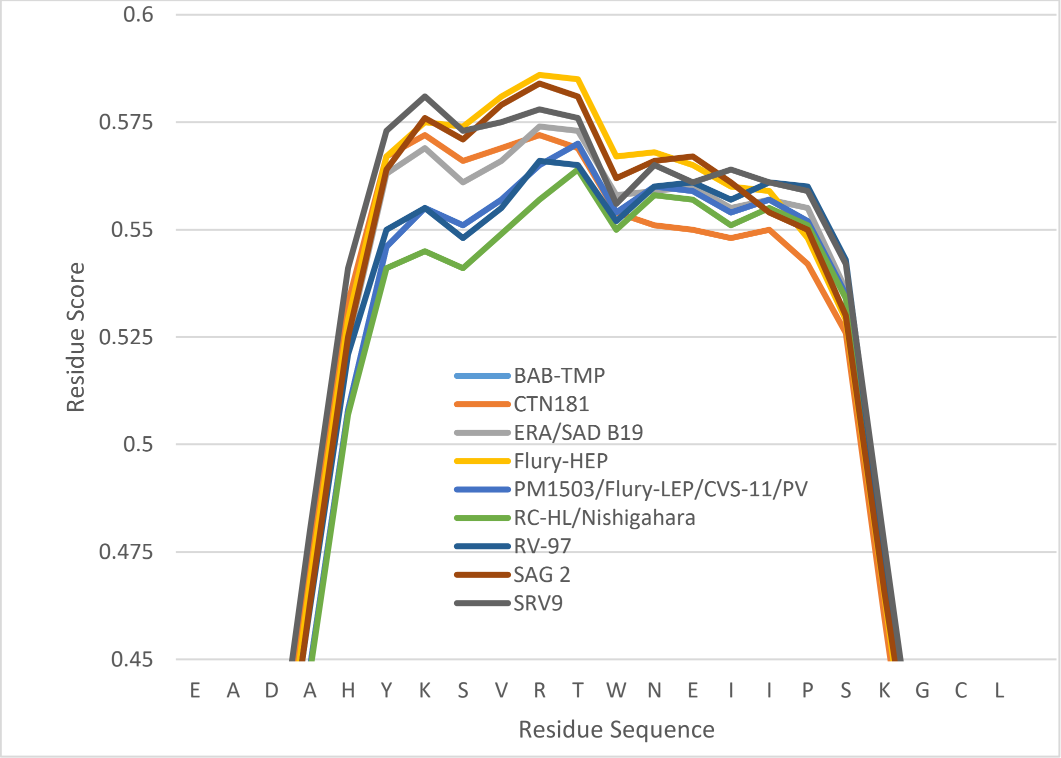


Figure S4. Linear B-cell epitope probability predicted with Bepipred 2.0 algorithm for Antigenic Site III
